# The Thousand Faces of Invasive Group A Streptococcal Infections: Update on Epidemiology, Symptoms, and Therapy

**DOI:** 10.3390/children11040383

**Published:** 2024-03-22

**Authors:** Stefania Mercadante, Andrea Ficari, Lorenza Romani, Maia De Luca, Costanza Tripiciano, Sara Chiurchiù, Francesca Ippolita Calo Carducci, Laura Cursi, Martina Di Giuseppe, Andrzej Krzysztofiak, Stefania Bernardi, Laura Lancella

**Affiliations:** 1 Infectious Disease Unit, Bambino Gesù Children’s Hospital, IRCCS, 00165 Rome, Italy; stefania.mercadante@opbg.net (S.M.); lorenza.romani@opbg.net (L.R.); costanza.tripiciano@opbg.net (C.T.); sara.chiurchiu@opbg.net (S.C.); stefania.bernardi@opbg.net (S.B.); laura.lancella@opbg.net (L.L.); 2 Residency School of Pediatrics, University of Rome Tor Vergata, 00133 Rome, Italy; andrea.ficari@opbg.net

**Keywords:** invasive *Streptococcus pyogenes* infection, β-lactam antibiotics, children

## Abstract

Invasive infections caused by *Streptococcus pyogfenes* (iGAS), commonly known as Group A Streptococcus, represent a significant public health concern due to their potential for rapid progression and life-threatening complications. Epidemiologically, invasive GAS infections exhibit a diverse global distribution, affecting individuals of all ages with varying predisposing factors. The pathogenesis of invasive GAS involves an array of virulence factors that contribute to tissue invasion, immune evasion, and systemic dissemination. In pediatrics, in the last few years, an increase in iGAS infections has been reported worldwide becoming a challenging disease to diagnose and treat promptly. This review highlights the current knowledge on pathogenesis, clinical presentations, and therapeutic approaches for iGAS in children.

## 1. Introduction

*Streptococcus pyogenes*, or group A Streptococcus (GAS), is a Gram-positive coccus responsible for both non-invasive—pharyngitis, scarlet fever, impetigo—as well as life-threatening invasive diseases defined as isolation of GAS from a normally sterile site of the body. In children, invasive GAS (iGAS) infection most commonly manifests as cellulitis, streptococcal toxic shock syndrome (STSS), necrotizing fasciitis (NF), and pneumonia [1]. Other forms of invasive disease include osteomyelitis, meningitis, bacteremia, arthritis, myositis, and endocarditis. GAS bacteremia usually arises as a consequence of a primary site of infection, most commonly skin and soft tissues. Alarmingly, the number of iGAS infections among children under 10 years old has risen in several European countries during the fall and winter of 2022, compared to previous years [2,3,4].

The factors responsible for the increase in the incidence of iGAS infections are not fully understood.

One of the hypotheses is the presence of a large group of susceptible individuals, particularly children, as a consequence of the pandemic restriction measures that have reduced the exposure to typical childhood infections with the subsequent lack of specific immunity to GAS—the so-called “immune debt”. To support this, a burden of respiratory tract infections caused by viruses such as Influenza Virus and Respiratory Syncytial Virus (RSV), was reported in the same period [5].

iGAS infections require hospitalization and can progress rapidly, leading to high mortality rates, particularly among young children, patients with comorbidities, and pregnant women.

A multitude of virulence factors characterize the highly pathogenic nature of GAS. The primary virulence factor of GAS is the M protein (encoded by the emm gene), an immunodominant surface antigen playing a central role in the organism’s capacity for colonization, evasion of phagocytosis, and invasion of sterile sites. Furthermore, similar to Staphylococcus aureus, GAS has the capability to produce exotoxins with superantigen properties. Among these exotoxins, Streptococcal pyrogenic exotoxin A (SpeA) is the most extensively studied, although various other pyrogenic exotoxins have been identified, such as SpeC, SpeG, SpeH, SpeI, SpeJ, SpeK, SpeL, SpeM, streptococcal mitogenic exotoxin Z (SMEZ), and streptococcal superantigen (SSA) [6].

Other secreted molecules believed to play a role in pathogenesis include the hemolytic toxins streptolysin O (SLO), streptolysin S (SLS), and enzymes such as hyaluronidase, streptokinase, nicotinamide-adenine dinucleotidase, and deoxyribonucleases [7].

This review aims to provide a comprehensive overview of the epidemiology, pathogenetic factors, clinical manifestations, diagnosis, treatment, and prevention strategies associated with iGAS infections in the pediatric population.

Post-streptococcal diseases, such as post-streptococcal glomerulonephritis (PSGN), acute rheumatic fever (ARF), and pediatric autoimmune neuropsychiatric disorders associated with streptococcal infections (PANDAS), primarily driven by an aberrant immune response following a GAS infection [8] will not be the subject of this review.

## 2. Epidemiology

The implementation of surveillance programs in many developed countries has led to the collection of data on the occurrence and seriousness of *S. pyogenes* infection, contributing to an improved understanding and control of these illnesses. Outbreaks of GAS infections may occur periodically in the general population or specific vulnerable groups, due to variation in circulating clones, emergence of novel strains, or host-related factors, for instance, decreased immunity or underlying medical conditions.

Sherwood et al., in a systematic review and meta-analyses published in 2022 calculated an incidence of iGAS infections in children (1–5 years old) of 0.22 per 1000 person-years in Low-Middle Income Countries (LMICs) and 0.05 per 1000 person-years in children for High Income Countries (HICs) [9]. Concerning newborns, the same meta-analysis computed an overall incidence of neonatal iGAS disease worldwide as 0.04 (95% CI 0.03–0.05) per 1000 live births, higher in LMICs (0.12 per 1000 live births) compared to HICs (0.02 per 1000 live births).

A 2005 global review estimated that 163,000 deaths each year worldwide were caused by iGAS disease, becoming an important cause of morbidity and mortality [10]. More recent data reported a pooled case fatality rate of 9% in children aged 0–5 years [9]. Comparing mortality rates between HICs and LMICs is challenging due to the scarcity of data available for LMICs.

From 2005 to 2019, increases in the incidence of iGAS infections have been described worldwide. The incidence rate in Ireland rose from 2.33–3.66 per 100,000 population to 0.8–1.65 per 100,000 population in 2012–2015 compared to the previous eight years [11]. A retrospective, multicentric study conducted in three University hospitals of the Brussels Capital Region showed an increase in iGAS diseases from 2.1 to 10.9/100,000 inhabitants between 2009 and 2019 [12]. During these years, a similar trend was observed in other European countries, such as Finland [13], Denmark [14], and Spain [15], as well as in other nations worldwide, from the United States [16] to Australia [17].

During the global COVID-19 pandemic, the containment measures adopted to restrict the spread of SARS-CoV-2 were responsible for the decreased incidence of respiratory infections and life-threatening invasive diseases worldwide [18]. Indeed, in 2020 and 2021 iGAS rates in children aged 2 to 17 were the lowest on record since 1997 [19].

In December 2022, an alert reporting an abnormal surge in both non-invasive and invasive Group A Streptococcus (GAS) diseases was published in the United Kingdom [3]. Similar concerns regarding heightened occurrences of GAS infections during 2022, particularly since September 2022, have also been reported by several other European countries (such as Ireland, France, the Netherlands, Denmark, and Sweden) [20,21]. Specifically, Spain experienced an almost four-fold rise in incidence during December 2022 compared to the months with the highest case numbers in 2019 [15]. Likewise, in the Netherlands in 2022, there was an over two-fold rise in the number of iGAS infections compared to pre-pandemic records, particularly notable in children under 5 years old [22].

In Denmark, since November 2022, case numbers of iGAS infections started to surge, reaching a peak in January 2023, showing a 3.5-fold increase compared to the rates observed in 2018/19. The most notable relative increase was particularly evident among children under 5 years old when compared to the pre-COVID-19 restriction period [23]. Notably, this heightened occurrence of iGAS cases happened concurrently with the weeks when respiratory syncytial virus (RSV) and other respiratory viruses were most prevalent [15]. A recent report from the UK showed that up to 60% of iGAS cases in children had a concurrent viral respiratory tract infection [5] as a consequence of reduced immunity to both GAS and common viral respiratory infections as a result of COVID-19 isolation measures. Furthermore, viral coinfection could have promoted iGAS infections due to various mechanisms, including local depletion of immune cells, suppression of immune cell response, local inflammation, and epithelial damage [24,25,26].

With the increase in absolute numbers of iGAS cases in children, several deaths among children under the age of 10 were reported within a brief period. Interestingly, within the clustered cases reported by the Spanish multicenter network for analyzing iGAS in Spain (PedGAS-net) in late 2022, there was a notable rise in ICU admissions of iGAS compared to pre-pandemic years [15].

## 3. Emm Types and Other Virulence Factors

Epidemiologically, Group A Streptococcus can be categorized into over 220 emm types [27], determined by the gene sequence of the amino terminal of the M protein.

These emm types exhibit distinct patterns of distribution, both regionally and globally [28].

The M protein, a fibrillar protein structured as a dimeric coil extending from the bacterial cell wall, is crucial for GAS virulence. Its primary contribution is attributed to the immune-modulatory effects it induces. It has the capability to directly bind different host components on the streptococcal surface, providing protection against both innate and adaptive immune responses [29]. The emm type is also an epidemiological marker that is used worldwide for characterizing GAS isolates, with an important role in antibiotic resistance studies and vaccine development [30].

Globally, emm types 1 (18.3%), 12 (11.1%), 28 (8.5%), 3 (6.9%), and 4 (6.9%) are responsible for approximately 40% of the disease burden [28].

The frequency of isolated emm types in different iGAS diseases generally corresponds to the reported rate in asymptomatic carriers within the same population.

However, a significant association has been described between certain emm types and specific disease manifestations.

Most research indicates that strains classified as emm 1 and emm3 types are linked with invasive disease, particularly with NF and STSS [31,32].

In 2009, a study conducted on 600 isolates obtained from iGAS in children across Europe, revealed that the most common emm types were emm1 (26%), emm12 (11%), emm4 and emm3 (10% each), and emm28 (7%) [33].

A French monocentric study conducted in a single tertiary care pediatric center yielded similar results with emm1 being the most prevalent, followed by emm12, emm3, and emm4 in iGAS [34].

Recently a significant contribution of emm 4 in iGAS was observed, notably increasing from 6–10% to 20% in children aged 0–5 years [4].

Superantigens play a pivotal role in determining the virulence of GAS. These bacterial toxins are known for their ability to bypass the normal intracellular antigen processing and presentation by the major histocompatibility complex (MHC) class II. By cross-linking of MHC class II molecules, superantigens induce a broad antigen-independent activation of T cells. This activation triggers the massive release of inflammatory cytokines such as interferon-gamma (IFN-γ), interleukin-1 (IL-1), and tumor necrosis factor-alpha (TNF-α). The overproduction of these cytokines can result in tissue damage, organ failure, and shock. Lintges et al. [7] have established an association between emm types and specific superantigen profiles, demonstrating a correlation between the presence of genes Spea1–Spea3 and the presence of the gene emm1.

Toxigenic M1UK, an emm1 sub-lineage initially identified in 2010 [35], recently emerged as the predominant cause of iGAS infections in numerous countries [36]. This type is characterized by a marked increase in the production of SpeA. During the recent outbreak described in 2022–2023, the emergence of another novel lineage, named M1DK, was observed and was characterized by the acquisition of a bacteriophage containing the exotoxin speC [37].

## 4. Clinical Features

iGAS infection exhibits a broad clinical spectrum and, usually, the presenting symptoms are non-specific, posing a diagnostic challenge for clinicians [38,39]. The two main points of entry for GAS are the respiratory tract and the skin. Various predisposing factors are associated with iGAS infection, such as other viral infections (e.g., influenza, varicella), skin disruption from trauma, immunodeficiency, malignant neoplasm, and age less than 1 year [1,40].

Streptococcal toxic shock syndrome (STSS) is a potentially life-threatening toxin-mediated condition. It is triggered by superantigens (such as exotoxins) and other virulence factors capable of activating the immune system by bypassing the typical sequence of antigen-mediated immune response.

The superantigen simultaneously interacts with T-cell receptor beta-chain variable regions and MHC class II, leading to the activation of a significant number of T cells in an antigen-independent manner. This process elicits a robust cytokine response from both T cells (lymphotoxin-α, IL-2, and IFN-γ) and antigen-presenting cells (TNF- α, IL-1, and IL-6). STSS often presents initially with flu-like symptoms, such as fever, chills, myalgia, nausea, and vomiting. These symptoms can rapidly progress to sepsis with hypotension, tachycardia, tachypnea, and signs and symptoms suggestive of organ failure [41].

**Figure 1:**
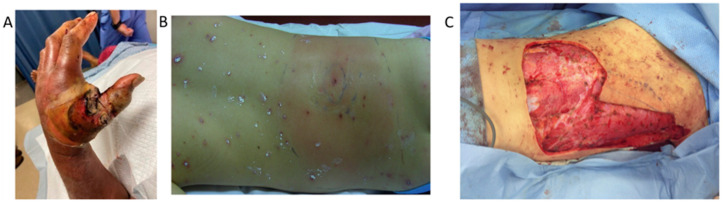
(**A**) Skin lesions of a patient presenting with NF, copyright by Szilagyi J et al., JPOSNA, 2023; Copyright 2023, owner’s Szilagyi, J., et al. [70] (**B**,**C**) Preoperative and intraoperatively findings of necrotizing fasciitis following varicella infection on the back of a 5-year-old boy; Copyright by Pfeifle VA et al., J.EPSC, 2017 Copyright 2017, owner’s Pfeifle, V.A. [71].

The outcome Is extensive tissue damage, disseminated intravascular thrombosis, and organ dysfunction [8]. The diagnostic criteria for probable and confirmed Streptococcal TSS were proposed by the Centers for Disease Control and Prevention (CDC) [41]. Streptococcal necrotizing fasciitis (NF) causes tissue damage through superantigens, which generally leads to a robust Th1 cytokine reaction, marked by elevated levels of TNF-β and IFN-γ, along with the presence of proinflammatory cytokines such as TNF-α and IL-1 [42]. In a single-center study, GAS was identified as the most frequently isolated organism in pediatric cases of NF followed by Staphylococcus aureus [43]. Approximately 40 to 50% of patients with necrotizing soft tissue infection will develop STSS [44,45]. The NF typically affects the extremities, with the lower extremity being more commonly involved than the upper extremity (Figure 1). The presentation is typically acute and characterized by localized symptoms such as pain (disproportionate to physical examination findings), swelling, redness, and warmth in the affected area [38]. Particularly in children, manifestations may encompass fever, tachycardia, leukocytosis, elevated creatine phosphokinase, hypoalbuminemia, hypocalcemia, and signs of coagulopathy. Within 24–72 h, there is a progression toward heightened inflammation, with the appearance of cutaneous findings such as hemorrhagic or serous bullae. In the subsequent days, the affected tissue, potentially extending to the muscle, becomes necrotic. To confirm the presence of the causative bacteria, obtaining culture and Gram stains from deep tissue specimens, or detecting positive blood cultures, is crucial. Radiographic imaging can be helpful in assessing the presence of necrotizing infection but should not be a reason to postpone surgical intervention. The most suitable initial radiographic imaging examination is a computed tomography (CT) scan [46]. The crucial measure to impede necrosis progression is prompt surgical debridement and antibiotic therapy [47]. The reported mortality rate in NF in pediatric cohorts is 5–15% [48,49], lower than that reported in adults [50].

*Streptococcus pyogenes* is the second leading cause of bacterial pneumonia after *Streptococcus pneumoniae* [51]. Interestingly, in the recent outbreak of iGAS infections, pneumonia has been described as the most frequent clinical manifestation, in contrast to previous reports [15]. Only 4.9% of patients with GAS pneumonia have positive blood cultures, which is lower compared to pneumonia caused by *Streptococcus pneumoniae* and *Staphylococcus aureus* [52]. Furthermore, several studies have demonstrated that patients with GAS pneumonia experience a more severe progression. They are at higher risk compared to those with pneumococcal infection of developing moderate-to-large pleural effusions, requiring mechanical ventilation, and enduring prolonged hospitalization [52].

Among the cerebral iGAS infections, the most common is meningitis, followed by cerebral abscess, subdural empyema, and subdural effusions [53]. However, meningitis caused by GAS is rare, accounting for less than 1% of all childhood bacterial meningitis [54] and fewer than 1–2% of iGAS disease cases in Europe and the United States [44,55]. Recently, van der Putten et al. reported an increase in GAS meningitis, particularly those caused by the emm1 type M1UK toxigenic variant in the Netherlands between 2022 and March 2023 [22]. Parameningeal infections, such as otitis media, sinusitis, and mastoiditis, are observed in 68% of intracranial iGAS infections [53,56]. Other potential routes of cerebral infection are penetrating head trauma, neurosurgical procedures, respiratory tract infections, and dental interventions. GAS meningitis is linked to a high case fatality rate, ranging from 15% to more than 40%, particularly when complicated by STSS [53,57]. A substantial incidence (up to 40%) of severe neurological sequelae has also been reported, especially in children [58].

Although S. Aureus remains the most common causative pathogen of musculoskeletal infections in children [59], septic arthritis and osteomyelitis caused by *S. pyogenes* have been reported, especially in older children [60,61].

According to published case studies, GAS is responsible for approximately 15–25% [62,63] of septic arthritis cases in children and around 7% of pediatric osteomyelitis cases [61,64].

Volske et al., employing a mice model for sepsis investigation, discovered the capability of Group A Streptococcus to infiltrate the articular microenvironment and showed that in vitro infection of fibroblast-like synoviocytes induces the expression of chemokines (Ccl2, Cxcl2), inflammatory cytokines (TNF, IL-6), and integrin ligands (ICAM-1, VCAM-1) and upregulates mediators affecting bone remodeling (Rankl) and cartilage integrity (Mmp13) [65].

In 90% of cases, septic arthritis affects lower limbs joints, with the hip being the most commonly involved, followed by the knee [66,67]. Regarding osteomyelitis, the most frequent sites are the tibia and femur [68,69].

The primary presentation of septic arthritis usually is joint pain, followed by symptoms such as fever, swelling, and restricted movement, accompanied by an elevation in CRP and erythrocyte sedimentation rate [66].

The standard diagnostic procedure for septic arthritis in children involves arthrocentesis [67] in order to identify GAS through culture, followed by standard imaging (X-ray, MRI) to rule out bone involvement.

## 5. Treatment

GAS universally retains sensitivity to β-lactam antibiotics, which remain the drugs of choice [72].

As patients with iGAS infection might not be easily discernible from bacterial sepsis syndromes caused due to other pathogens, initial treatment should involve broad-spectrum antibiotics, which can be subsequently tailored with penicillin or other β-lactams once iGAS infection is confirmed.

Specific considerations must be made for STSS and NF. In these cases, the therapy includes administration of a β-lactam combined with clindamycin. Clindamycin inhibits the synthesis of crucial virulence factors such as streptolysin S, M-protein, and streptococcal pyrogenic exotoxins, and could enhance GAS opsonization and phagocytosis through complement [8,73].

Resistance mechanisms to macrolide and lincosamide antibiotics are rising, leading to recurrent infections, treatment failure, and poor patient outcomes. Between 2011 and 2019, the US Centers for Disease Control and Prevention (CDC) reported a rise from 11.9% to 24.7% in iGAS isolates exhibiting non-susceptibility to erythromycin, and from 8.9% to 23.8% to clindamycin [19]. In Europe, resistance to clindamycin has been reported in 4–7% of iGAS isolates [12,74], although recently, alarming data from an Irish case study reported a resistance rate to clindamycin in invasive forms of 28% [75]. Resistance mechanisms include ribosomal target site modification mediated by erythromycin resistance methylase (Erm) proteins, conferring resistance to macrolides and lincosamides [76]. The mefA (macrolide efflux pump A) gene in GAS confers resistance to erythromycin and azithromycin [77]. Regional variations in resistance rates can be attributed to differences in the prevalence of mefA-expressing and erm-expressing isolates.

Linezolid also inhibits protein production by targeting the 50S ribosome subunit, representing a promising alternative adjunctive antitoxin antibiotic for iGAS NF and STSS. Invasive GAS strains show consistently high susceptibilities to linezolid, with a reported 100% susceptibility in the SENTRY dataset, encompassing 3893 US GAS clinical isolates from 2013–2020 [78]. Similar to clindamycin, linezolid diminishes GAS virulence, as demonstrated by in vitro studies [79,80]. However, the clinical evidence supporting linezolid for severe GAS infections is comparatively limited to that supporting clindamycin. No clinical trials have demonstrated that adjunctive linezolid reduces mortality or otherwise improves outcomes in iGAS infections. A small retrospective cohort study on severe GAS infections found comparable outcomes with adjunctive clindamycin and linezolid and favorable outcomes in iGAS infections treated with adjunctive linezolid have also been reported [81,82]. Linezolid presents potential advantages, including its universal susceptibility among GAS, lower risk of C. difficile infection compared with clindamycin, and concurrent coverage against MRSA. However, currently, there is insufficient evidence to demonstrate its superiority over clindamycin, especially in regions where GAS susceptibility to clindamycin remains high.

Furthermore, the emergence of subclinical β-lactam resistance in GAS continues to be a persistent concern. In streptococcal species, including GAS, penicillin resistance primarily arises from mutations in penicillin-binding proteins (PBPs), which are the target sites for β-lactam antibiotics. In 2020, two clinical *Streptococcus pyogenes* isolates belonging to the emm subtype emm43.4 with a pbp2x missense mutation (T553K) were identified, showing an eightfold reduction in susceptibility to both ampicillin and amoxicillin [83]. Another study examining 13,727 invasive GAS isolates in the United States from 2015 to 2021, found 388 PBP2x variants with elevated β-lactam MICs [84].

In addition to antibiotic therapy, the cornerstones of managing invasive forms are hemodynamic support and source control. In the case of STSS, fluid administration and hemodynamic support are crucial to maintaining organ perfusion [85].

Polyvalent intravenous immunoglobulins (IVIG) are commonly employed as supplementary therapy for STSS, aiming to neutralize superantigens and improve bacterial elimination by facilitating opsonization of Group A Streptococcus (GAS) for phagocytosis. Several studies, including non-randomized trials [86,87,88] and a small randomized control trial [89], suggest that IVIG as adjunctive therapy could reduce mortality in STSS. The suggested protocol consists of 1 g/kg on day 1, followed by 0.5 g/kg on days 2 and 3.

The use of parenteral non-steroidal anti-inflammatory drugs to treat fever is thought to increase the risk of systemic complications in iGAS due to the masking of symptoms, thus contributing to the delay of an appropriate treatment [90]. There is no evidence supporting the use of steroids in iGAS.

Research involving the application of anti-TNF antibodies and IL-1 inhibitors in animal models of STSS has shown encouraging outcomes [91,92,93], but further studies are needed.

## 6. Prevention

Despite extensive research ongoing since the 1940s, currently no licensed vaccine for GAS is available. The main obstacle is represented by the absence of established human immune indicators of protection that could facilitate the narrowing down of candidate antigens [94]. Therefore, there is an urgent need to develop a vaccine against Strep A to prevent streptococcal infection and to reduce associated morbidity and mortality. The World Health Organization Product Development for Vaccines Advisory Committee (PD-VAC) and the Coalition to Advance New Vaccines Against Group A Streptococcus (CANVAS) are endorsing its development [95]. Efforts to develop a GAS vaccine have primarily centered on the M protein, given its high immunogenicity and role in triggering strain-specific immunity against GAS [77,96,97,98,99,100]. The majority of existing vaccine candidates are still undergoing preclinical investigation, with only two actively being assessed in human trials [96,101].

## 7. Contact Prophylaxis

Contacts of iGAS disease have an increased risk of contracting the infection. Outbreaks of iGAS infections within family clusters, school children, and communities are well described [102,103,104]. The UK guidelines recommend antibiotic prophylaxis to all women from ≥37 weeks of pregnancy up to 28 days of giving birth who are close contacts of the iGAS case. Chemoprophylaxis is also recommended to neonates up to 28 days after birth (where the mother or any close contact develops iGAS infection), to people aged >75 years old, and to individuals who develop chickenpox with active lesions within 7 days prior to diagnosis of iGAS infection in the index case or within 48 h after commencing antibiotics for the iGAS case if exposure is ongoing. The regimen is based on 10-day penicillin V or, as a second line, 10-day clarithromycin for children and 10-day clarithromycin or 5-day azithromycin for adults (10-day erythromycin for pregnant or post-partum) [105]. Following the UK guidelines, antibiotic prophylaxis should be administered within 24 h after the eligible contacts are identified, and not beyond 10 days after iGAS diagnosis in the index case [105]. The Canadian public health authorities recommend contact prophylaxis for all close contacts of severe iGAS [106], whilst the US Centers for Disease Control and Prevention recommends against routine administration of chemoprophylaxis for household contacts unless they are at increased risk for invasive GAS infection [107].

## 8. Conclusions

Invasive GAS infections are severe and often life-threatening. The increased incidence of iGAS infection in children demands the development of clear guidelines on how to promptly diagnose and treat the disease. The available evidence supports the need for the early initiation of supportive care and broad empiric antibacterial therapy that includes β-lactam antibiotics and the association of clindamycin due to its anti-toxin capacity. Early administration of IVIG is recommended, especially if there is evidence of STSS, along with debridement of necrotic tissue in cases of NF. A heightened awareness of the clinical spectrum, risk factors, and advancements in diagnostic and therapeutic modalities is crucial for improving outcomes in affected children.

## Data Availability

Not applicable.
